# Di­chlorido­bis­[2-(pyridin-2-yl-κ*N*)-1*H*-benzimidazole-κ*N* ^3^]nickel(II) monohydrate

**DOI:** 10.1107/S2414314620000401

**Published:** 2020-01-28

**Authors:** Connor S. MacNeil, Aloice O. Ogweno, Stephen O. Ojwach, Paul G. Hayes

**Affiliations:** a Department of Chemistry and Biochemistry, University of Lethbridge, 4401, University Drive Lethbridge, AB, T1K 3M4, Canada; b Department of Chemistry, South Eastern University of Kenya, Kitui, Kenya; c School of Chemistry & Physics, University of KwaZulu-Natal, Scottsville, Pietermaritzburg, South Africa; University of Neuchâtel, Switzerland

**Keywords:** crystal structure, nickel(II) complex, 2-(pyridin-2-yl)-1*H*-benzimidazole, hydrogen bonding, C—H⋯π inter­actions, transfer hydrogenation

## Abstract

In the title complex, [NiCl_2_(C_12_H_9_N_3_)_2_]·H_2_O, a divalent nickel atom is coordinated by two 2-(pyridin-2-yl)-1*H*-benzimidazole ligands in a slightly distorted octa­hedral environment defined by four N donors of two *N*,*N*′-chelating ligands, along with two *cis*-oriented anionic chloride donors. The title complex crystallized with a water mol­ecule disordered over two positions. In the crystal, a combination of O—H⋯Cl, O—H.·O and N—H⋯Cl hydrogen bonds, together with C—H⋯O, C—H⋯Cl and C—H⋯π inter­actions, links the complex mol­ecules and the water mol­ecules to form a supra­molecular three-dimensional framework. The title complex is isostructural with the cobalt(II) dichloride complex reported previously [Das *et al.* (2011 ▸). *Org. Biomol. Chem*. **9**, 7097–7107].

## Abstract 2

The crystal structure of the nickel(II) dichloride complex of the ligand 2-(pyridin-2-yl)-1*H*-benzimidazole is described.

## Graphical abstract

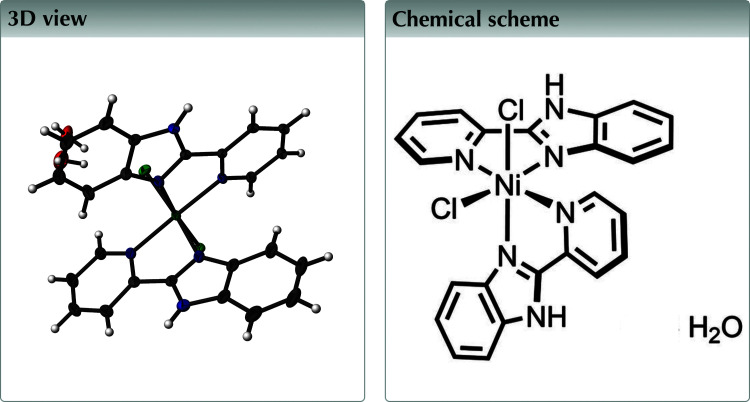

## Structure description

Transition-metal-catalyzed transfer hydrogenation (TH) is an effective method of reducing ketones to the corresponding secondary alcohols (Zhu *et al.*, 2014 ▸). Generally, the method is operationally simple, selective, and sources hydrogen from alcohols, thus avoiding high pressures of H_2_ gas (Zhu *et al.*, 2014 ▸). Several transition-metal complexes have been studied in catalytic TH and have been used on laboratory and industrial scales. Complexes of precious metals (Rh, Ir, and Ru) have been the preferred catalysts for TH owing to their high activity and commercial availability (Raja *et al.*, 2012 ▸; Wang *et al.*, 2015 ▸; Li *et al.*, 2015 ▸). With growing concern surrounding the economic and environmental impact of using precious metals in chemistry, a renewed inter­est in Earth-abundant metal catalysis has prompted our research into TH catalysts featuring first-row transition metals, such as iron, cobalt, or nickel (Morris, 2009 ▸; Garduño & García, 2017 ▸; Abubakar *et al.*, 2018 ▸; Chen *et al.*, 2010 ▸). Recognizing that nickel(II) complexes of chiral bis­(phosphines) have been utilized in asymmetric TH, we turned our attention to nickel(II) complexes of the commercially available ligand 2-(pyridin-2-yl)-1*H*-benzimidazole.

**Figure 1:**
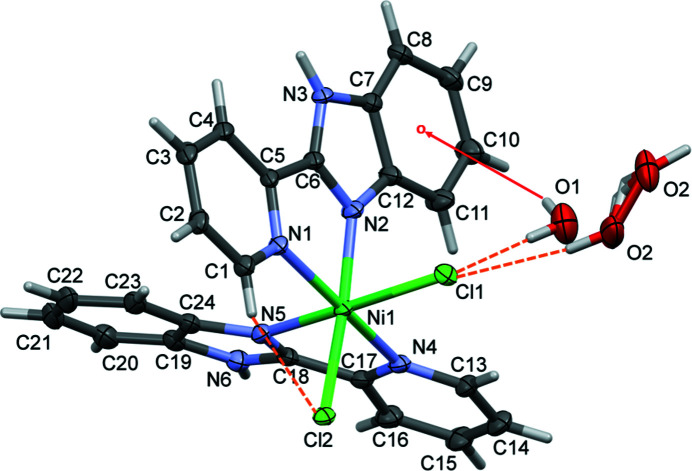
The mol­ecular structure of the title complex, with atom labeling. Displacement ellipsoids are drawn at the 50% probability level. Hydrogen bonds are shown as orange dashed lines and the O—H⋯π inter­action as a red arrow (Table 1 ▸).

The asymmetric unit of the title complex consists of a Ni^II^ ion coordinated by two 2-(pyridin-2-yl)-1*H*-benzimidazole ligands bound in a κ^2^-*N*,*N* arrangement, along with two *cis*-oriented anionic chloride donors (Fig. 1 ▸). The complex crystallized as a monohydrate with the water mol­ecule disordered over two sites (Fig. 1 ▸). The metal center adopts a slightly distorted octa­hedral geometry. The pyridyl N-donor atoms are *trans*-disposed [N1—Ni1—N4 = 170.66 (8)°], while the chloride ligands are *cis*-disposed [Cl2—Ni1—Cl1 = 93.04 (2)°]. The disordered water mol­ecules are linked to the complex mol­ecule by O—H⋯Cl hydrogen bonds, and water H atom H2*B* is directed to the centroid of the C7–C12 ring (Fig. 1 ▸, Table 1 ▸).

**Figure 2:**
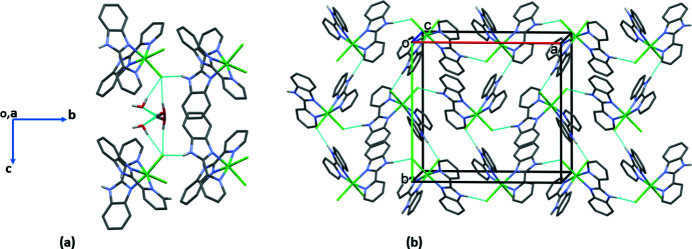
Hydrogen-bonding networks involving, (*a*) the discorded water mol­ecule, and (*b*) the N—H⋯Cl hydrogen bonds. For clarity, only the H atoms involved in hydrogen bonding (dashed lines; Table 1 ▸) have been included.

**Figure 3:**
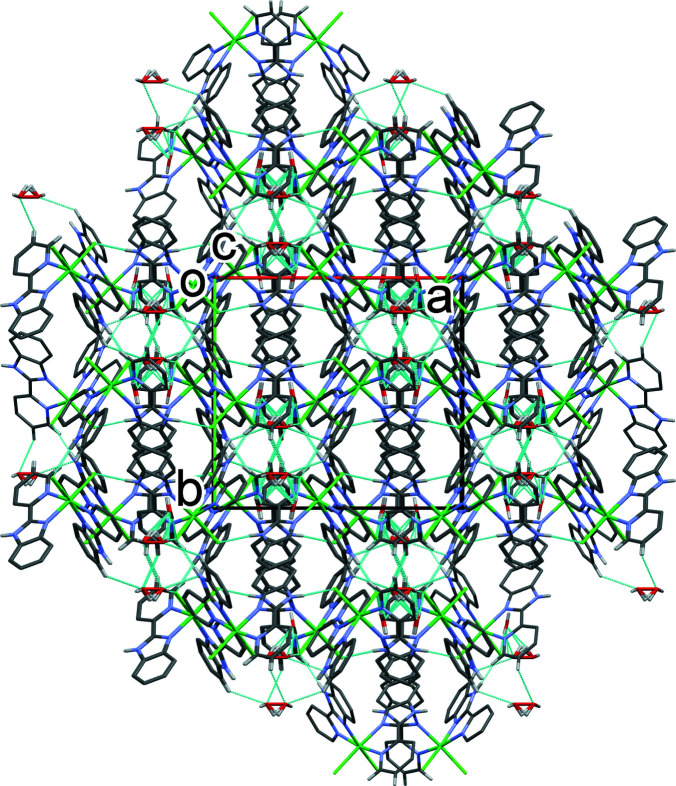
A view along the *c* axis of the crystal packing of the title complex. For clarity, only the H atoms involved in hydrogen bonding (dashed lines; Table 1 ▸) have been included.

In the crystal, extensive hydrogen bonding is observed involving the disordered water mol­ecule, the ligand NH groups and the chloride ions (Fig. 2 ▸ *a* and 2*b* and Table 1 ▸). The result is the formation of a supra­molecular three-dimensional network (Fig. 3 ▸). There are also C—H⋯O and C—H⋯π inter­actions present (Table 1 ▸) consolidating the packing.

**Figure 4:**
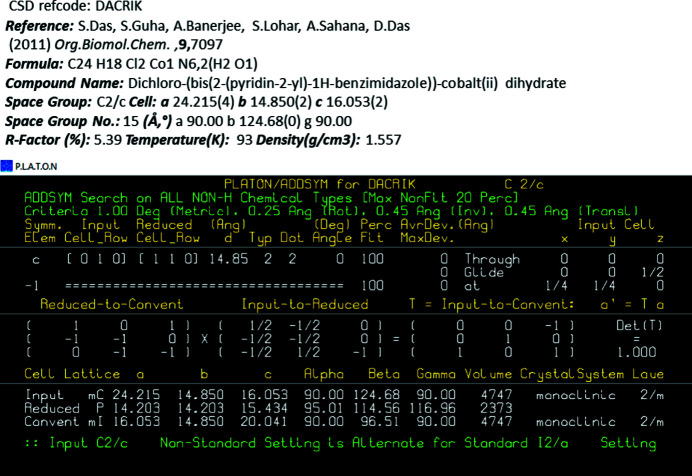
A view of the ADDSYM (*PLATON*; Spek, 2020 ▸) transformation of the cell dimensions of the isostructural compound di­chlorido­bis­[2-(pyridin-2-yl)-1*H*-benzimidazole]­cobalt(II) monohydrate (CSD refcode DACRIK; Das *et al.*, 2011 ▸).

A search of the Cambridge Structural Database (CSD, Version 5.40, May 2019; Groom *et al.*, 2016 ▸) revealed that the title compound is isostructural with the cobalt(II) complex di­chlorido­bis-[2-(pyridin-2-yl)-1*H*-benzimidazole]­cobalt(II) monohydrate (CSD refcode DACRIK; Das *et al.*, 2011 ▸). The later was reported in space group *C*2/*c* but transformation of the unit cell gives space group *I*2/*a* (ADDSYMM in *PLATON*; Spek, 2020 ▸) with almost identical cell parameters to those of the title complex – see Fig. 4 ▸.

## Synthesis and crystallization

**Figure 5:**
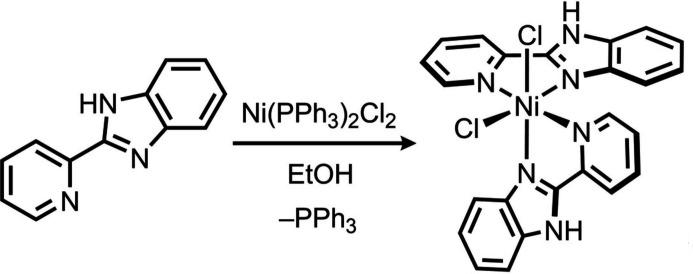
Reaction scheme for the synthesis of the title complex.

The reaction scheme for the synthesis of the title complex is given in Fig. 5 ▸. A solution of 2-(pyridin-2-yl)-1*H*-benzimidazole (0.15 g, 0.78 mmol) in ethanol (5 ml) was added dropwise to a stirring ethano­lic solution of bis­(tri­phenyl­phosphine)nickel(II) dichloride (0.50 g, 0.76 mmol). The mixture was stirred at room temperature for 24 h. The resulting mixture was concentrated and the product isolated by addition of diethyl ether (5 ml) giving a light-brown solid. Yield: 0.27 g (68%). Analysis calculated for C_24_H_18_Cl_2_N_6_Ni: C, 55.43; H, 3.49; N, 16.16%. Found: C, 55.23; H, 3.59; N, 16.25%. Light-blue plate-like crystals, suitable for X-ray diffraction analysis, were obtained by slow evaporation of a concentrated ethanol solution.

## Refinement

Crystal data, data collection and structure refinement details are summarized in Table 2 ▸. The complex crystallized as a monohydrate with the water mol­ecule disordered over two sites (O1 and O2); occupancies fixed at 0.5 each.

## Supplementary Material

Crystal structure: contains datablock(s) Global, I. DOI: 10.1107/S2414314620000401/su4175sup1.cif

Structure factors: contains datablock(s) I. DOI: 10.1107/S2414314620000401/su4175Isup2.hkl

CCDC reference: 1946553

Additional supporting information:  crystallographic information; 3D view; checkCIF report

## Figures and Tables

**Table 1:** Hydrogen-bond geometry (Å, °) *Cg*1, *Cg*2, *Cg*3, *Cg*4 and *Cg*5 are the centroids of the C7–C12, N5/N6/C18/C19/C24, N1/C1–C5, N4/C13–C17 and C19–C24 rings, respectively.

*D*—H⋯*A*	*D*—H	H⋯*A*	*D*⋯*A*	*D*—H⋯*A*
C1—H1⋯Cl2	0.95	2.75	3.378 (2)	124
O1—H1*A*⋯Cl1	0.85	2.37	3.221 (4)	174
O2—H2*B*⋯Cl1	0.85	2.41	3.239 (4)	165
O2—H2*A*⋯O1^i^	0.85	1.97	2.806 (6)	167
N3—H3⋯Cl2^ii^	0.88	2.29	3.162 (2)	171
N6—H6⋯Cl1^iii^	0.88	2.23	3.069 (2)	160
C2—H2⋯O2^iv^	0.95	2.56	3.400 (5)	147
C20—H20⋯O2^iii^	0.95	2.54	3.317 (5)	139
O1—H1*B*⋯*Cg*1	0.85	3.11	3.869 (3)	150
C3—H3*A*⋯*Cg*5^ii^	0.95	2.97	3.738 (3)	139
C8—H8⋯*Cg*2^v^	0.95	2.69	3.579 (3)	155
C9—H9⋯*Cg*5^v^	0.95	2.88	3.542 (3)	128
C11—H11⋯*Cg*4	0.95	2.93	3.810 (3)	155
C23—H23⋯*Cg*3	0.95	2.94	3.733 (3)	142

Symmetry codes: (i) 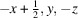 ; (ii) 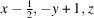 ; (iii) 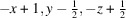 ; (iv) 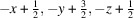 ; (v) 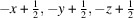 .

**Table 2:** Experimental details

Crystal data	
Chemical formula	[NiCl_2_(C_12_H_9_N_3_)_2_]·H_2_O
*M*_r_	538.07
Crystal system, space group	Monoclinic, *I*2/*a*
Temperature (K)	100
*a*, *b*, *c* (Å)	15.9019 (6), 14.7008 (7), 20.0039 (7)
β (°)	95.924 (4)
*V* (Å^3^)	4651.4 (3)
*Z*	8
Radiation type	Mo *K*α
μ (mm^−1^)	1.10
Crystal size (mm)	0.21 × 0.15 × 0.1

Data collection	
Diffractometer	Rigaku Oxford Diffraction SuperNova, Dual, Cu at zero, Pilatus 200/300K
Absorption correction	Multi-scan (*CrysAlis PRO*; Rigaku OD, 2015 ▸)
*T*_min_, *T*_max_	0.785, 1.000
No. of measured, independent and observed [*I* > 2σ(*I*)] reflections	29773, 6268, 5296
*R*_int_	0.046
(sin θ/λ)_max_ (Å^−1^)	0.734

Refinement	
*R*[*F* ^2^ > 2σ(*F* ^2^)], *wR*(*F* ^2^), *S*	0.043, 0.113, 1.06
No. of reflections	6268
No. of parameters	322
H-atom treatment	H-atom parameters constrained
	
Δρ_max_, Δρ_min_ (e Å^−3^)	1.16, −0.64

Computer programs: *CrysAlis PRO* (Rigaku OD, 2015 ▸), *SHELXT* (Sheldrick, 2015*a* ▸), *SHELXL* (Sheldrick, 2015*b* ▸), *OLEX2* (Dolomanov *et al.*, 2009 ▸) and *publCIF* (Westrip, 2010 ▸)’.

## full crystallographic data

### Crystal data

**Table:**

[NiCl_2_(C_12_H_9_N_3_)_2_]·H_2_O	*F*(000) = 2208
*M**_r_* = 538.07	*D*_x_ = 1.537 Mg m^−^^3^
Monoclinic, *I*2/*a*	Mo *K*α radiation, λ = 0.71073 Å
*a* = 15.9019 (6) Å	Cell parameters from 13739 reflections
*b* = 14.7008 (7) Å	θ = 3.8–30.9°
*c* = 20.0039 (7) Å	µ = 1.10 mm^−^^1^
β = 95.924 (4)°	*T* = 100 K
*V* = 4651.4 (3) Å^3^	Plate, clear light blue
*Z* = 8	0.21 × 0.15 × 0.1 mm

### Data collection

**Table:**

Rigaku Oxford Diffraction SuperNova, Dual, Cu at zero, Pilatus 200/300K diffractometer	5296 reflections with *I* > 2σ(*I*)
ω scans	*R*_int_ = 0.046
Absorption correction: multi-scan (CrysAlisPro; Rigaku OD, 2015)	θ_max_ = 31.4°, θ_min_ = 3.4°
*T*_min_ = 0.785, *T*_max_ = 1.000	*h* = −22→21
29773 measured reflections	*k* = −19→19
6268 independent reflections	*l* = −29→26

### Refinement

**Table:**

Refinement on *F*^2^	Primary atom site location: dual
Least-squares matrix: full	Secondary atom site location: difference Fourier map
*R*[*F*^2^ > 2σ(*F*^2^)] = 0.043	Hydrogen site location: mixed
*wR*(*F*^2^) = 0.113	H-atom parameters constrained
*S* = 1.06	*w* = 1/[σ^2^(*F*_o_^2^) + (0.0511*P*)^2^ + 11.2375*P*] where *P* = (*F*_o_^2^ + 2*F*_c_^2^)/3
6268 reflections	(Δ/σ)_max_ = 0.001
322 parameters	Δρ_max_ = 1.16 e Å^−^^3^
0 restraints	Δρ_min_ = −0.64 e Å^−^^3^

### Special details

**Table:**

Geometry. All esds (except the esd in the dihedral angle between two l.s. planes) are estimated using the full covariance matrix. The cell esds are taken into account individually in the estimation of esds in distances, angles and torsion angles; correlations between esds in cell parameters are only used when they are defined by crystal symmetry. An approximate (isotropic) treatment of cell esds is used for estimating esds involving l.s. planes.
Refinement. The O-, N- and C-bound H atoms were included in calculated positions and treated as riding on the parent atom: O—H = 0.85 Å, N—H = 0.88 Å, C—H = 0.95 Å with *U*_iso_(H) = 1.5*U*_eq_(O) and 1.2*U*_eq_(N,*C*).

### Fractional atomic coordinates and isotropic or equivalent isotropic displacement parameters (Å^2^)

**Table:**

	*x*	*y*	*z*	*U*_iso_*/*U*_eq_	Occ. (<1)
Ni1	0.41587 (2)	0.53396 (2)	0.26342 (2)	0.01946 (9)	
Cl2	0.51535 (3)	0.65004 (4)	0.30400 (2)	0.02048 (12)	
Cl1	0.34436 (3)	0.63357 (5)	0.17809 (3)	0.02851 (14)	
N1	0.32601 (11)	0.56738 (14)	0.32888 (9)	0.0206 (4)	
N4	0.50259 (11)	0.47857 (14)	0.20265 (9)	0.0223 (4)	
N3	0.19362 (11)	0.38608 (14)	0.25224 (9)	0.0214 (4)	
H3	0.1459	0.3800	0.2704	0.026*	
N5	0.47605 (11)	0.43649 (14)	0.32845 (9)	0.0216 (4)	
N2	0.32190 (11)	0.43767 (14)	0.23554 (9)	0.0230 (4)	
N6	0.54931 (12)	0.30631 (15)	0.33216 (10)	0.0251 (4)	
H6	0.5796	0.2610	0.3188	0.030*	
C1	0.33068 (14)	0.63587 (16)	0.37299 (11)	0.0220 (4)	
H1	0.3771	0.6767	0.3740	0.026*	
C6	0.25743 (13)	0.44376 (16)	0.27227 (10)	0.0197 (4)	
C2	0.27009 (14)	0.64957 (17)	0.41747 (11)	0.0239 (5)	
H2	0.2741	0.6996	0.4477	0.029*	
C13	0.52147 (14)	0.50999 (18)	0.14323 (12)	0.0260 (5)	
H13	0.4931	0.5627	0.1250	0.031*	
C7	0.21767 (13)	0.33846 (16)	0.19770 (11)	0.0220 (4)	
C5	0.25879 (13)	0.51099 (16)	0.32589 (10)	0.0200 (4)	
C3	0.20350 (14)	0.58805 (18)	0.41653 (11)	0.0251 (5)	
H3A	0.1628	0.5939	0.4479	0.030*	
C4	0.19672 (13)	0.51814 (17)	0.36966 (11)	0.0232 (5)	
H4	0.1509	0.4764	0.3676	0.028*	
C12	0.29871 (14)	0.37186 (17)	0.18758 (11)	0.0239 (5)	
C17	0.54399 (13)	0.40452 (16)	0.22848 (11)	0.0229 (4)	
C19	0.52027 (14)	0.31581 (18)	0.39437 (11)	0.0266 (5)	
C18	0.52220 (13)	0.38021 (17)	0.29551 (11)	0.0229 (4)	
C24	0.47421 (14)	0.39799 (17)	0.39175 (11)	0.0247 (5)	
C8	0.17716 (14)	0.27144 (18)	0.15768 (12)	0.0273 (5)	
H8	0.1232	0.2488	0.1658	0.033*	
C9	0.21903 (16)	0.23914 (19)	0.10533 (12)	0.0305 (5)	
H9	0.1928	0.1942	0.0761	0.037*	
C16	0.60480 (15)	0.35909 (18)	0.19622 (12)	0.0285 (5)	
H16	0.6329	0.3071	0.2159	0.034*	
C10	0.29967 (16)	0.2715 (2)	0.09461 (13)	0.0335 (6)	
H10	0.3266	0.2477	0.0582	0.040*	
C14	0.58124 (16)	0.46814 (19)	0.10720 (13)	0.0320 (6)	
H14	0.5929	0.4915	0.0648	0.038*	
C11	0.34100 (15)	0.3368 (2)	0.13516 (13)	0.0319 (6)	
H11	0.3960	0.3572	0.1279	0.038*	
C23	0.43824 (16)	0.42902 (19)	0.44841 (11)	0.0290 (5)	
H23	0.4070	0.4842	0.4476	0.035*	
C22	0.45004 (18)	0.3760 (2)	0.50589 (12)	0.0345 (6)	
H22	0.4270	0.3959	0.5453	0.041*	
C20	0.53109 (16)	0.2614 (2)	0.45225 (13)	0.0335 (6)	
H20	0.5614	0.2057	0.4534	0.040*	
C21	0.49486 (17)	0.2940 (2)	0.50755 (12)	0.0362 (6)	
H21	0.5006	0.2596	0.5479	0.043*	
C15	0.62325 (16)	0.3922 (2)	0.13411 (13)	0.0320 (5)	
H15	0.6644	0.3627	0.1105	0.038*	
O1	0.3180 (3)	0.5134 (3)	0.0429 (2)	0.0440 (10)	0.5
H1A	0.3268	0.5413	0.0802	0.066*	0.5
H1B	0.3085	0.4575	0.0502	0.066*	0.5
O2	0.2882 (3)	0.6487 (3)	0.01818 (18)	0.0429 (10)	0.5
H2A	0.2602	0.6019	0.0042	0.064*	0.5
H2B	0.3113	0.6391	0.0578	0.064*	0.5

### Atomic displacement parameters (Å^2^)

**Table:**

	*U*^11^	*U*^22^	*U*^33^	*U*^12^	*U*^13^	*U*^23^
Ni1	0.01005 (13)	0.03529 (17)	0.01374 (13)	−0.00391 (10)	0.00448 (10)	−0.00355 (11)
Cl2	0.0109 (2)	0.0340 (3)	0.0169 (2)	−0.00172 (18)	0.00350 (17)	−0.00279 (19)
Cl1	0.0169 (2)	0.0491 (4)	0.0188 (2)	−0.0029 (2)	−0.00126 (19)	0.0018 (2)
N1	0.0130 (8)	0.0355 (10)	0.0141 (8)	−0.0013 (7)	0.0049 (6)	−0.0016 (7)
N4	0.0129 (8)	0.0379 (11)	0.0165 (8)	−0.0066 (7)	0.0038 (7)	−0.0047 (8)
N3	0.0098 (7)	0.0387 (11)	0.0162 (8)	−0.0036 (7)	0.0033 (6)	0.0001 (8)
N5	0.0154 (8)	0.0346 (10)	0.0153 (8)	−0.0064 (7)	0.0036 (7)	−0.0023 (7)
N2	0.0133 (8)	0.0401 (11)	0.0166 (8)	−0.0052 (8)	0.0059 (7)	−0.0046 (8)
N6	0.0167 (8)	0.0376 (11)	0.0208 (9)	−0.0022 (8)	0.0007 (7)	−0.0010 (8)
C1	0.0172 (10)	0.0334 (12)	0.0158 (9)	−0.0009 (8)	0.0035 (8)	−0.0014 (8)
C6	0.0114 (9)	0.0319 (11)	0.0159 (9)	−0.0005 (8)	0.0020 (7)	0.0000 (8)
C2	0.0196 (10)	0.0384 (13)	0.0143 (9)	0.0055 (9)	0.0048 (8)	−0.0004 (9)
C13	0.0179 (10)	0.0420 (13)	0.0192 (10)	−0.0054 (9)	0.0064 (8)	−0.0015 (9)
C7	0.0141 (9)	0.0351 (12)	0.0163 (9)	−0.0028 (8)	0.0000 (8)	0.0011 (9)
C5	0.0113 (9)	0.0368 (12)	0.0120 (8)	0.0018 (8)	0.0020 (7)	0.0007 (8)
C3	0.0149 (9)	0.0445 (14)	0.0166 (9)	0.0053 (9)	0.0049 (8)	0.0013 (9)
C4	0.0111 (9)	0.0423 (13)	0.0163 (9)	0.0005 (8)	0.0022 (8)	0.0036 (9)
C12	0.0160 (10)	0.0385 (13)	0.0172 (9)	−0.0054 (9)	0.0025 (8)	−0.0057 (9)
C17	0.0129 (9)	0.0362 (12)	0.0196 (10)	−0.0059 (8)	0.0021 (8)	−0.0051 (9)
C19	0.0190 (10)	0.0422 (13)	0.0178 (10)	−0.0096 (9)	−0.0019 (8)	−0.0011 (9)
C18	0.0128 (9)	0.0383 (12)	0.0173 (9)	−0.0046 (8)	−0.0002 (8)	−0.0024 (9)
C24	0.0174 (10)	0.0386 (13)	0.0176 (10)	−0.0094 (9)	−0.0009 (8)	−0.0009 (9)
C8	0.0180 (10)	0.0414 (14)	0.0216 (10)	−0.0077 (9)	−0.0025 (9)	−0.0010 (10)
C9	0.0255 (11)	0.0430 (14)	0.0219 (11)	−0.0073 (10)	−0.0036 (9)	−0.0077 (10)
C16	0.0179 (10)	0.0418 (14)	0.0264 (11)	−0.0009 (9)	0.0053 (9)	−0.0049 (10)
C10	0.0252 (12)	0.0514 (16)	0.0245 (11)	−0.0056 (11)	0.0050 (9)	−0.0140 (11)
C14	0.0236 (12)	0.0501 (16)	0.0242 (11)	−0.0064 (11)	0.0123 (10)	−0.0039 (11)
C11	0.0204 (11)	0.0509 (15)	0.0260 (12)	−0.0089 (10)	0.0098 (9)	−0.0165 (11)
C23	0.0253 (11)	0.0434 (14)	0.0182 (10)	−0.0138 (10)	0.0021 (9)	−0.0041 (10)
C22	0.0358 (14)	0.0520 (16)	0.0158 (10)	−0.0193 (12)	0.0023 (10)	−0.0037 (10)
C20	0.0270 (12)	0.0454 (15)	0.0263 (12)	−0.0106 (11)	−0.0057 (10)	0.0043 (11)
C21	0.0362 (14)	0.0533 (17)	0.0174 (10)	−0.0191 (12)	−0.0048 (10)	0.0042 (11)
C15	0.0201 (11)	0.0511 (15)	0.0268 (12)	−0.0027 (10)	0.0114 (9)	−0.0093 (11)
O1	0.057 (3)	0.048 (2)	0.0260 (19)	0.009 (2)	−0.0008 (18)	0.0042 (17)
O2	0.052 (3)	0.057 (3)	0.0188 (17)	−0.022 (2)	0.0026 (17)	−0.0022 (16)

### Geometric parameters (Å, º)

**Table:**

Ni1—Cl2	2.4101 (6)	C3—C4	1.388 (3)
Ni1—Cl1	2.4394 (6)	C4—H4	0.9500
Ni1—N1	2.0937 (18)	C12—C11	1.401 (3)
Ni1—N4	2.0949 (19)	C17—C18	1.463 (3)
Ni1—N5	2.099 (2)	C17—C16	1.388 (3)
Ni1—N2	2.0918 (19)	C19—C24	1.411 (4)
N1—C1	1.336 (3)	C19—C20	1.403 (3)
N1—C5	1.349 (3)	C24—C23	1.398 (3)
N4—C13	1.338 (3)	C8—H8	0.9500
N4—C17	1.347 (3)	C8—C9	1.382 (4)
N3—H3	0.8800	C9—H9	0.9500
N3—C6	1.351 (3)	C9—C10	1.405 (4)
N3—C7	1.383 (3)	C16—H16	0.9500
N5—C18	1.326 (3)	C16—C15	1.393 (4)
N5—C24	1.390 (3)	C10—H10	0.9500
N2—C6	1.325 (3)	C10—C11	1.378 (3)
N2—C12	1.385 (3)	C14—H14	0.9500
N6—H6	0.8800	C14—C15	1.382 (4)
N6—C19	1.378 (3)	C11—H11	0.9500
N6—C18	1.356 (3)	C23—H23	0.9500
C1—H1	0.9500	C23—C22	1.386 (4)
C1—C2	1.392 (3)	C22—H22	0.9500
C6—C5	1.457 (3)	C22—C21	1.398 (4)
C2—H2	0.9500	C20—H20	0.9500
C2—C3	1.391 (3)	C20—C21	1.385 (4)
C13—H13	0.9500	C21—H21	0.9500
C13—C14	1.394 (3)	C15—H15	0.9500
C7—C12	1.413 (3)	O1—H1A	0.8505
C7—C8	1.386 (3)	O1—H1B	0.8503
C5—C4	1.389 (3)	O2—H2A	0.8507
C3—H3A	0.9500	O2—H2B	0.8498
			
Cl2—Ni1—Cl1	93.04 (2)	C5—C4—H4	120.9
N1—Ni1—Cl2	95.15 (5)	C3—C4—C5	118.1 (2)
N1—Ni1—Cl1	89.87 (5)	C3—C4—H4	120.9
N1—Ni1—N4	170.66 (8)	N2—C12—C7	108.96 (19)
N1—Ni1—N5	93.99 (7)	N2—C12—C11	131.4 (2)
N4—Ni1—Cl2	91.27 (5)	C11—C12—C7	119.6 (2)
N4—Ni1—Cl1	96.57 (6)	N4—C17—C18	113.3 (2)
N4—Ni1—N5	78.99 (8)	N4—C17—C16	123.1 (2)
N5—Ni1—Cl2	91.90 (5)	C16—C17—C18	123.4 (2)
N5—Ni1—Cl1	173.44 (6)	N6—C19—C24	105.9 (2)
N2—Ni1—Cl2	174.23 (5)	N6—C19—C20	131.6 (3)
N2—Ni1—Cl1	87.19 (6)	C20—C19—C24	122.5 (2)
N2—Ni1—N1	79.09 (7)	N5—C18—N6	113.1 (2)
N2—Ni1—N4	94.43 (7)	N5—C18—C17	119.9 (2)
N2—Ni1—N5	88.32 (8)	N6—C18—C17	126.8 (2)
C1—N1—Ni1	126.52 (15)	N5—C24—C19	108.8 (2)
C1—N1—C5	118.82 (18)	N5—C24—C23	130.9 (2)
C5—N1—Ni1	114.63 (15)	C23—C24—C19	120.2 (2)
C13—N4—Ni1	127.08 (17)	C7—C8—H8	121.6
C13—N4—C17	118.3 (2)	C9—C8—C7	116.8 (2)
C17—N4—Ni1	114.60 (15)	C9—C8—H8	121.6
C6—N3—H3	126.5	C8—C9—H9	119.4
C6—N3—C7	106.94 (17)	C8—C9—C10	121.2 (2)
C7—N3—H3	126.5	C10—C9—H9	119.4
C18—N5—Ni1	111.00 (15)	C17—C16—H16	121.1
C18—N5—C24	105.3 (2)	C17—C16—C15	117.9 (2)
C24—N5—Ni1	142.33 (16)	C15—C16—H16	121.1
C6—N2—Ni1	112.26 (15)	C9—C10—H10	118.9
C6—N2—C12	105.40 (18)	C11—C10—C9	122.2 (2)
C12—N2—Ni1	142.08 (15)	C11—C10—H10	118.9
C19—N6—H6	126.6	C13—C14—H14	120.6
C18—N6—H6	126.6	C15—C14—C13	118.9 (2)
C18—N6—C19	106.8 (2)	C15—C14—H14	120.6
N1—C1—H1	118.8	C12—C11—H11	121.3
N1—C1—C2	122.4 (2)	C10—C11—C12	117.4 (2)
C2—C1—H1	118.8	C10—C11—H11	121.3
N3—C6—C5	126.71 (19)	C24—C23—H23	121.4
N2—C6—N3	113.18 (19)	C22—C23—C24	117.2 (3)
N2—C6—C5	120.04 (19)	C22—C23—H23	121.4
C1—C2—H2	120.9	C23—C22—H22	119.0
C3—C2—C1	118.3 (2)	C23—C22—C21	122.0 (2)
C3—C2—H2	120.9	C21—C22—H22	119.0
N4—C13—H13	118.8	C19—C20—H20	122.1
N4—C13—C14	122.3 (2)	C21—C20—C19	115.9 (3)
C14—C13—H13	118.8	C21—C20—H20	122.1
N3—C7—C12	105.53 (19)	C22—C21—H21	118.9
N3—C7—C8	131.7 (2)	C20—C21—C22	122.1 (2)
C8—C7—C12	122.7 (2)	C20—C21—H21	118.9
N1—C5—C6	113.64 (18)	C16—C15—H15	120.3
N1—C5—C4	122.5 (2)	C14—C15—C16	119.5 (2)
C4—C5—C6	123.9 (2)	C14—C15—H15	120.3
C2—C3—H3A	120.1	H1A—O1—H1B	109.4
C4—C3—C2	119.8 (2)	H2A—O2—H2B	109.5
C4—C3—H3A	120.1		

### Hydrogen-bond geometry (Å, º)

*Cg*1, *Cg*2, *Cg*3, *Cg*4 and *Cg*5 are the centroids of the C7–C12, N5/N6/C18/C19/C24, N1/C1–C5, N4/C13–C17 and C19–C24 rings, respectively.

**Table:**

*D*—H···*A*	*D*—H	H···*A*	*D*···*A*	*D*—H···*A*
C1—H1···Cl2	0.95	2.75	3.378 (2)	124
O1—H1*A*···Cl1	0.85	2.37	3.221 (4)	174
O2—H2*B*···Cl1	0.85	2.41	3.239 (4)	165
O2—H2*A*···O1^i^	0.85	1.97	2.806 (6)	167
N3—H3···Cl2^ii^	0.88	2.29	3.162 (2)	171
N6—H6···Cl1^iii^	0.88	2.23	3.069 (2)	160
C2—H2···O2^iv^	0.95	2.56	3.400 (5)	147
C20—H20···O2^iii^	0.95	2.54	3.317 (5)	139
O1—H1*B*···*Cg*1	0.85	3.11	3.869 (3)	150
C3—H3*A*···*Cg*5^ii^	0.95	2.97	3.738 (3)	139
C8—H8···*Cg*2^v^	0.95	2.69	3.579 (3)	155
C9—H9···*Cg*5^v^	0.95	2.88	3.542 (3)	128
C11—H11···*Cg*4	0.95	2.93	3.810 (3)	155
C23—H23···*Cg*3	0.95	2.94	3.733 (3)	142

Symmetry codes: (i) −*x*+1/2, *y*, −*z*; (ii) *x*−1/2, −*y*+1, *z*; (iii) −*x*+1, *y*−1/2, −*z*+1/2; (iv) −*x*+1/2, −*y*+3/2, −*z*+1/2; (v) −*x*+1/2, −*y*+1/2, −*z*+1/2.
