# Di­chlorido­bis­[2-(pyridin-2-yl-κ*N*)-1*H*-benzimidazole-κ*N* ^3^]nickel(II) monohydrate. Corrigendum

**DOI:** 10.1107/S2414314620006884

**Published:** 2020-05-29

**Authors:** Connor S. MacNeil, Aloice O. Ogweno, Stephen O. Ojwach, Paul G. Hayes

**Affiliations:** a Department of Chemistry and Biochemistry, University of Lethbridge, 4401, University Drive Lethbridge, AB, T1K 3M4, Canada; b Department of Pure and Applied Sciences, Technical University of Mombasa, Mombasa, Kenya; c School of Chemistry & Physics, University of KwaZulu-Natal, Scottsville, Pietermaritzburg, South Africa

## Abstract

In the paper by MacNeil *et al.* [ *IUCrData*, (2020), **5**, x200040], the address of the second author is incorrect.

## Abstract 2

Corrigendum to *IUCrData* (2020), **5**, x200040.

In the paper by MacNeil *et al.* (2020 ▸), the address of the second author, Aloice O. Ogweno, should be ‘Department of Pure and Applied Sciences, Technical University of Mombasa, Mombasa, Kenya’, as given above.
